# The Dallas Meeting of the State Medical Association

**Published:** 1902-05

**Authors:** 


### TEXAS MEDICAL JOURNAL.

AUSTIN, TEXAS.

A MONTHLY JOURNAL OF MEDICINE AND SURGERY.

EDITED AND PUBLISHED BY
F. E. DANIEL, M. D.

ASSOCIATE EDITOR:
WITTEN BOOTH RUSS, M. D.,
San Antonio, Texas.

Published Monthly at Austin, Texas. Subscription price $1.00 a year in advance.

Eastern Representative: John Guy Monihan, St. Paul Building, 220 Broadway, New York City.

Official organ of the State Association of Health Officers, the West Texas Medical Association, the Houston District Medical Association, the Austin District Medical Society, the Brazos Valley Medical Association, the Galveston County Medical Society, and several others.

### THE DALLAS MEETING OF THE STATE MEDICAL ASSOCIATION.

In a few days after this shall have been read by our subscribers the big annual meeting of the State Medical Association will take place (May 6th, 7th, 8th, 9th). It will not only be one of the largest gatherings of Texas physicians ever held, but will be one of the most important meetings of the Association. There is to be a complete revolution in the organic law, a shaking up of the dry bones, a change in the policy which for twenty years has kept the membership at a standstill, notwithstanding the large acquisitions made every year. The JOURNAL has for years advocated some such change, and has repeatedly pointed out that the "fall off" every year, nearly or quite offsets the gains in membership, and has stated the reasons for it. The membership is scarcely more than it was in 1884. Now, under the new arrangement every reputable physician in the State will find it to his vital interest to become a member and remain a member. He will have to be a member of his local society in order to be eligible; and the time is not far distant when every man who is not a member will be a marked man. The people will soon recognize that he is not a member because he *can't be;* and thus the sheep will be separated from the goats, and the goats will be permitted to go to grass, or hunt pastures new. In this issue readers will find a circular letter from the State Association to local societies in affiliation, which should be carefully read and studied before the reader goes to Dallas. It will let him know "what's up," and it is necessary to know it so as to save trouble and time in explanations after he gets there. I also publish the program in full. It will be found very attractive. For the information of those who intend joining I will state here, in reply to numerous inquiries received by mail, that until the new constitution is adopted, and it will be adopted at Dallas and go into effect from and after its passage, applicants will be received in the usual way. One must carry with him his diploma, or his certificate from the State Board of Medical Examiners; write out an application on blanks furnished, and secure the endorsement of two members. The fee stands as it is until changed, $5.00. Next year one cannot join unless he be a member of his local society.

